# Translation and adaptation of the Radiotherapy Edema Rating Scale to Brazilian Portuguese^☆^

**DOI:** 10.1016/j.bjorl.2017.03.014

**Published:** 2017-05-09

**Authors:** Débora dos Santos Queija, Lica Arakawa-Sugueno, Bruna Mello Chamma, Marco Aurélio Vamondes Kulcsar, Rogério Aparecido Dedivitis

**Affiliations:** a Universidade de São Paulo (USP), Faculdade de Medicina, Curso de Pós-Graduação em Fisiopatologia Experimental, São Paulo, SP, Brazil; b Universidade de São Paulo (USP), Faculdade de Medicina, Ciências, São Paulo, SP, Brazil; c Universidade Braz Cubas, Mogi das Cruzes, SP, Brazil; d Universidade de São Paulo (USP), Faculdade de Medicina, Departamento de Cirurgia, São Paulo, SP, Brazil; e Instituto do Câncer do Estado de São Paulo (ICESP), Serviço de Cirurgia de Cabeça e Pescoço, São Paulo, SP, Brazil; f Universidade de São Paulo (USP), Faculdade de Medicina, Grupo de Tumores de Laringe e Hipofaringe do Serviço de Cirurgia de Cabeça e Pescoço, São Paulo, SP, Brazil

**Keywords:** Edema, Head and neck neoplasms, Radiotherapy, Pharynx, Larynx, Edema, Neoplasias de cabeça e pescoço, Radioterapia, Faringe, Laringe

## Abstract

**Introduction:**

Internal lymphedema is one of the sequelae of head and neck cancer treatment that can lead to varying degrees of swallowing, speech, and respiration alterations. The Radiotherapy Edema Rating Scale, developed by Patterson et al., is a tool used to evaluate pharyngeal and laryngeal edema.

**Objective:**

To translate into Brazilian Portuguese, to culturally adapt and test this scale in patients undergoing treatment for head and neck cancer.

**Methods:**

The process followed the international guidelines and translation steps by two head and neck surgeons and back-translation performed independently by two North-American natives. The final version of the test was evaluated based on the assessment of 18 patients by two head and neck surgeons and two speech therapists using the scales in Brazilian Portuguese.

**Results:**

The translation and cultural adaptation were satisfactorily performed by the members of the committee in charge.

**Conclusion:**

The translation and adaptation into Brazilian Portuguese of the Radiotherapy Edema Rating Scale was successfully performed and showed to be easy to apply.

## Resumo

**Introdução:**

O linfedema interno é uma das sequelas do tratamento para o câncer de cabeça e pescoço, podendo levar a alterações de grau variado na deglutição, voz e respiração. A Escala do Edema da Radioterapia (*Radiotherapy Edema Rating Scale*), elaborada por Patterson et al., é uma ferramenta de avaliação do edema de faringe e laringe.

**Objetivo:**

Traduzir, para o português brasileiro, adaptar culturalmente e testar esta escala em pacientes submetidos ao tratamento para o câncer de cabeça e pescoço.

**Método:**

O processo seguiu as diretrizes internacionais e as etapas de tradução por dois cirurgiões de cabeça e pescoço e a retrotradução de forma independente por dois nativos norte-americanos. O teste da versão final para avaliação foi realizado a partir da avaliação de 18 pacientes por dois médicos cirurgiões de cabeça e pescoço e duas fonoaudiólogas por meio da aplicação das escalas em português.

**Resultados:**

A tradução e adaptação cultural foram executadas satisfatoriamente pelos membros do comitê responsável.

**Conclusão:**

A tradução e adaptação da Escala do Edema da Radioterapia para o português foi bem sucedida e de fácil aplicação.

## Introduction

The head and neck encompasses an extensive lymphatic network and more than 300 lymph nodes (one-third of the lymph nodes in the body).^1^ The treatment for head and neck cancer involves multimodal therapies that result in increased survival rates; however, they are accompanied by the risk of secondary complications, such as secondary lymphedema. The tumor, surgery, and radiotherapy can break down lymphatic structures and block lymph flow, resulting in soft tissue edema. Muscle contraction and soft tissue compression facilitate lymphatic flow through movement. However, the damage caused by surgery and radiotherapy adversely modifies this mechanism, leading to reduced movement and lymph flow.^1^, ^2^, ^3^, ^4^

Lymphatic dysfunction occurs when any lymphatic structure or that surrounding soft tissue is damaged by cancer and its treatment, limiting the capacity of the lymphatic system to transport the lymph volume carried to the tissues. Lymphedema is a swelling that develops during a period of at least three months after head and neck cancer treatment, beyond the period when acute edema occurs.^5^, ^6^, ^7^

When the lymphedema develops, the lymphatic system may be able to repair or compensate for the damage done, resulting in visible swelling reduction. If the damage is severe or there is no intervention, the accumulated protein-rich lymphatic fluid can trigger a chronic inflammatory response, resulting in a fibrosclerotic process wherein fatty or fibrous tissues may develop.^1^, ^7^

Head and neck cancer lymphedema may affect external (face, submental and neck) and internal structures (upper aerodigestive tract, tongue, epiglottis) or both (compound). Internal lymphedema may impair chewing, swallowing, speech, and voice.^8^ Both types can progress over time and, when identified and treated early, swelling regression and prevention of late effects, such as fibrosis, may be the result.^9^ Therefore, it is important to assess for lymphedema as part of the clinical routine of the evaluation of head and neck cancer patients.^1^, ^3^, ^6^, ^8^, ^10^, ^11^

Few measures to evaluate edema and lymphedema have been developed over the last few years. Concern about these aspects has been increasing in the last decade, aiming to identify, and monitor the evolution of alterations and treatment results.^11^, ^12^, ^13^, ^14^

The Radiotherapy Edema Rating Scale, developed by Patterson et al.,^15^ is the most comprehensive tool that evaluates and stages, in a simple and objective manner, 11 structures and two spaces of the pharynx and larynx through endoscopy. The scale showed good intra-rater (Kappa = 0.84) and moderate inter-rater (Kappa = 0.54) reliability.

The aim of this study is to carry out the translation of the Radiotherapy Edema Rating Scale into Brazilian Portuguese and its cross-cultural adaptation.

## Methods

This study represents the initial phase of the clinical study project, approved by the Ethics Committee of the institution where it was performed, under number 528/14. To develop the work using the scale, permission was granted by the author, who authorized the translation.

Because this scale evaluates structures strictly related to anatomy, the translation was performed by two head and neck surgeons with experience in head and neck edema and lymphedema, who were proficient in the English language, based on the Nomina Anatomica.^16^ The process was based on international guidelines.

Subsequently, a consensus developed between the translators regarding a Brazilian Portuguese version and subsequent back-translation performed by two native speakers of the English language, independently. Following that, the comparison of the back-translation with the original scale was performed, analyzing aspects related to conceptual, semantic and content equivalence and later creation of a translated version by the committee, which consisted of the translators and back-translators.

Eighteen patients submitted to surgical and/or radio-chemotherapy treatment were evaluated by nasoendoscopy, which was recorded on DVD for further evaluation by the committee.

The final version was applied by four health professionals (two head and neck surgeons and two speech therapists, with broad experience in head and neck cancer and interpretation of videoendoscopic images of the pharynx and larynx). Due to similar interpretations, the evaluators achieved consensus.

## Results

The translation of the Radiotherapy Edema Rating Scale (Table 1) was performed independently by two head and neck surgeons proficient in the English language.^15^

**Table 1:** Radiotherapy Edema Rating Scale (original tool in the English language).

	Rating of edema			
Structures	Normal	Mild	Moderate	Severe
Base of tongue				
Posterior pharyngeal wall				
Epiglottis				
Pharyngoepiglottic folds				
Aryepiglottic folds				
Interarytenoid space				
Cricopharyngeal prominence				
Arytenoids				
False vocal folds				
True vocal folds				
Anterior commissure				

Spaces	Normal	Mildly reduced	Moderately reduced	Severely reduced
Vallecullae				
Pyriform sinus				

The two translations (Table 2, Table 3) were analyzed jointly by the two translators, who reached a consensus for its final version in Brazilian Portuguese (Table 4). There was a question regarding the term cricopharyngeal prominence, which in Portuguese refers to the cricopharyngeal bar, an alteration related to the anatomy of patients submitted to total laryngectomy. To clarify this doubt, we contacted the author and asked whether the term would correspond to the cricopharyngeal prominence. The author confirmed our hypothesis.

**Table 2:** Radiotherapy Edema Rating Scale (Translator A).

	Classificação do edema			
Estruturas	Normal	Discreto	Moderado	Intenso
Base da língua				
Parede posterior de faringe				
Epiglote				
Pregas faringo-epiglóticas				
Pregas ariepiglóticas				
Membrana interaritenóidea				
Área pós-cricóide				
Aritenóides				
Bandas ventriculares				
Pregas vocais				
Comissura anterior				

Espaços	Normal	Redução discreta	Redução moderada	Redução intensa
Valécula				
Seios piriformes

**Table 3:** Radiotherapy Edema Rating Scale (Translator B).

	Classificação do edema			
Estruturas	Normal	Discreto	Moderado	Severo
Base da língua				
Parede posterior de faringe				
Epiglote				
Pregas faringo-epiglóticas				
Pregas ariepiglóticas				
Espaço interaritenóideo				
Área retrocricóidea				
Aritenóides				
Pregas vestibulares				
Pregas vocais				
Comissura anterior				

Espaços	Normal	Discretamente reduzida	Moderadamente reduzida	Severamente reduzida
Valécula				
Seios piriformes

**Table 4:** Final version of the Radiotherapy Edema Rating Scale (consensus between translators A and B).

	Classificação do edema			
Estruturas	Normal	Discreto	Moderado	Severo
Base da língua				
Parede posterior de faringe				
Epiglote				
Pregas faringo-epiglóticas				
Pregas ariepiglóticas				
Espaço interaritenóideo				
Área retrocricóidea				
Aritenóides				
Pregas vestibulares				
Pregas vocais				
Comissura anterior				

Espaços	Normal	Discretamente reduzida	Moderadamente reduzida	Severamente reduzida
Valécula				
Seios piriformes				

Based on this last version, the back-translation was carried out independently by two bilingual translators. In the case of the term that raised doubts in the translators, it was understood in the back-translation as post-cricoid area. Thus, the versions were similar to each other without any impairment to the original version. The committee chose to retain the original version, with the term cricopharyngeal prominence (Table 5).

**Table 5:** Radiotherapy edema rating (independent back-translation).

	Rating of edema			
Structures	Normal	Mild	Moderate	Severe
Base of the tongue				
Posterior pharyngeal wall				
Epiglottis				
Pharyngoepiglottic folds				
Aryepiglottic folds				
Interarytenoid space				
Cricopharyngeal prominence				
Arytenoids				
False vocal folds				
Vocal folds				
Anterior commissure				

Spaces	Normal	Slightly reduced	Moderately reduced	Severely reduced
Valleculla				
Pyriform sinus				

The authors chose to translate the pyriform sinus structure as *seio piriforme* because, although the Nomina indicates the term “pyriform recess,” the term piriform sinus is widely used.

The examinations were then performed by a head and neck surgeon in the 18 patients recruited for the study.

The tool was applied by the group consisting of two head and neck surgeons and two speech therapists (who had experience in interpreting nasoendoscopy results) in a consensus, to the 18 patients at the institution where the study was carried out (Table 6, Table 7). Because this is a scale that evaluates anatomical structures, we did not observe any difficulties in understanding and applying the tool.

**Table 6:** Demographic, clinical and treatment characteristics.

Variable	Category	*n*
Age	Min.–max.	36–82
	Median	60
	Mean ± standard deviation	61.22 ± 11.39

Gender	Female	6
	Male	12

Tumor location	Mouth	7
	Oropharynx	5
	Larynx	1
	Infraglottic	1
	Thyroid	1
	Face	2
	Occult primary tumor	1

Staging	Tx	1
	T1b	1
	T2	10
	T3	2
	T4	2
	N0	10
	N1	2
	N2	1
	N2a	2
	N2b	1

Treatment	Surgery	8
	Surgery + radiotherapy	4
	Surgery + radio-chemo	5
	Radio-chemotherapy	1

Neck dissection	No	3
	Yes	15

Type of neck dissection	Supraomohyoid	9
	Radical	3
	Modified radical	1
	Jugular	1
	Selective	1

Radiotherapy	Min.–max.	3150–7000
	Median	1575
	Mean ± standard deviation	3186 ± 3292.57

Time until the end of treatment (months)	Min.–max.	3–40
	Median	6.5
	Mean ± standard deviation	11.94 ± 12.12

Alcoholism	No	18
	Yes	–

Smoking	No	16
	Yes	2

Tracheostomy	No	17
	Yes	1

Nasogastric tube	No	17
	Yes	1

**Table 7:** Distribution of radiotherapy edema classification.

Patients	Structures											Spaces	
	BT	PPW	E	PEF	AEF	IS	CPP	A	FVF	VF	AC	V	PS
1	1	0	2	0	1	2	2	3	2	0	0	2	1
2	0	0	0	0	0	0	0	0	0	0	0	1	0
3	0	0	0	0	1	2	0	2	0	0	0	0	0
4	0	0	0	0	0	1	0	1	0	1	0	0	0
5	0	1	1	0	2	2	2	3	1	0	0	0	2
6	0	0	0	0	0	2	2	0	0	0	0	0	0
7	0	0	0	0	0	0	0	0	0	0	0	0	0
8	1	0	1	1	0	2	2	2	0	0	0	1	0
9	0	1	0	0	0	0	0	0	0	0	0	0	0
10	0	0	1	0	2	2	2	2	0	0	0	0	0
11	0	0	1	0	0	1	2	1	0	0	0	0	0
12	0	0	0	0	0	0	0	0	0	0	0	0	0
13	1	2	1	0	2	2	1	2	0	0	0	1	1
14	1	0	1	0	2	2	2	2	0	0	0	1	0
15	0	2	1	2	1	2	2	2	0	0	0	0	1
16	0	0	0	0	0	0	0	0	0	0	0	0	0
17	0	0	1	2	2	2	2	2	0	0	0	0	2
18	2	2	2	2	3	3	3	3	0	0	0	0	3

BT, base of the tongue; PPW, posterior pharyngeal wall; E, epiglottis; PEF, pharyngoepiglottic folds; AEF, aryepiglottic folds; IS, Interarytenoid space; CPP, cricopharyngeal prominence; A, arytenoids; FVF, false vocal folds; VF, vocal folds; AC, anterior commissure; V, valleculla; PS, pyriform sinus; degree of edema of structures: 0, normal; 1, mild edema; 2, moderate edema; 3, severe edema; degree of space reduction: 0, normal; 1, mildly reduced; 2, moderately reduced; 3, severely reduced.

## Discussion

The techniques of edema and lymphedema assessment through images are tools that offer a more accurate choice of the structures involved both with the disease and the treatment. The evaluation of internal edema secondary to treatment in head and neck cancer is a tool that can contribute not only to its diagnosis but also to its evolution.

Other modalities such as lymphoscintigraphy, magnetic resonance imaging, computed tomography, ultrasonography, and fluorescence imaging, scarcely mentioned in the literature of the head and neck region, are also used in addition to the laryngological evaluation using the Radiotherapy Edema Rating Scale. The Patterson Scale can be easily applied in clinical practice, since laryngological examination is part of the routine evaluation and follow-up of patients with head and neck cancer.^1^, ^14^, ^17^, ^18^, ^19^, ^20^, ^21^, ^22^

Another possibility is to verify the association of swallowing and voice alterations with the presence of pharyngeal and laryngeal edema, which can be better quantified using the Radiotherapy Edema Rating Scale. The association between internal edema and swallowing and breathing alterations and their impact on quality of life using this scale identified a strong correlation between edema severity, especially in the region of the aryepiglottic folds, pharyngoepiglottic folds, epiglottis, arytenoids, and pyriform sinus with swallowing symptoms, mainly of solid consistency. When compared to patients without internal edema, the impact on function and quality of life was more evident.^10^, ^22^

Damage to the lymphatic tissues can lead to lymphedema and fibrosis, which may manifest as early or late effects of head and neck cancer treatment. Lymphedema and fibrosis are not static processes. Lymphedema is associated with ongoing inflammation resulting in progressive fibrosis and adipose tissue deposition. With the development of fibrofatty tissue, manual lymphatic drainage and compression therapy may be less effective. Therefore, the evaluation of treatment effects may facilitate an earlier approach aiming to avoid or minimize these alterations.^23^

The Radiotherapy Edema Rating Scale is indicated by several authors as a valid tool for the characterization of edema after head and neck cancer treatment.^1^, ^2^, ^12^, ^18^, ^22^

## Conclusions

The translation of the Radiotherapy Edema Rating Scale into Brazilian Portuguese was compatible with the original. The tool is accessible and easy to interpret for health professionals experienced in the evaluation and treatment of head and neck cancer.

## Conflicts of interest

The authors declare no conflicts of interest.
